# Tubulocystic Carcinoma of the Bile Duct

**DOI:** 10.1155/2018/2304610

**Published:** 2018-04-01

**Authors:** Masahiro Takeuchi, Yoshitaka Sakamoto, Hirotsugu Noguchi, Sohsuke Yamada, Keiji Hirata

**Affiliations:** ^1^Department of Surgery 1, School of Medicine, University of Occupational and Environmental Health, 1-1 Iseigaoka, Yahatanishi-ku, Kitakyushu-shi, Fukuoka 807-0804, Japan; ^2^Department of Surgery, Moji Medical Center, 3-1 Higashiminatomachi, Moji-ku, Kitakyushu-shi, Fukuoka 801-8502, Japan; ^3^Department of Pathology, School of Medicine, University of Occupational and Environmental Health, 1-1 Iseigaoka, Yahatanishi-ku, Kitakyushu-shi, Fukuoka 807-0804, Japan; ^4^Department of Pathology and Laboratory Medicine, Kanazawa Medical University, 1-1 Uchinada, Ishikawa 920-0293, Japan

## Abstract

Tubulocystic carcinoma of the bile duct is extremely rare and has not been reported in the literature. We reported a case of cystic neoplasm of the liver with distinct histopathological features that could not be clearly classified as of either mucinous or intraductal papillary neoplasm. A 68-year-old Japanese patient had a multicystic biliary tumor within the liver. This tumor was detected on follow-up of polymyalgia rheumatica. The exophytic, multicystic, 35 × 50 mm mass was composed of complex tubulocystic structures. We initially suspected cystadenocarcinoma of the liver and performed radical operation. However, pathology ultimately showed it to be very rare tubulocystic carcinoma that derived from the bile duct. We reviewed the literature and describe the process of our differential diagnosis.

## 1. Introduction

Tubulocystic carcinoma is one of subtypes of kidney cancer, but it is a rare structure in kidney cancer [1]. On the other hand, intrahepatic bile duct carcinoma is a malignant tumor that develops from the intrahepatic bile duct epithelium, including the glands [2, 3]. Intrahepatic cholangiocarcinoma is the second most common type of hepatocellular carcinoma in primary malignant liver tumors. Many clinicopathologic studies on hepatocellular carcinoma have been published, but intrahepatic bile duct carcinoma remains unstudied in many respects, including its pathology form. We herein report that cystic tumor of biliary tract with feature mimicking a subtype of renal carcinoma.

## 2. Case Report

A 68-year-old man was referred to our department for the evaluation and treatment of a liver mass detected on inspections that were performed due to poor control of polymyalgia rheumatica. Abdominal ultrasound (US) and computed tomography (CT) revealed a solid 3.0 cm mass, which was suspected to be cystadenocarcinoma, in the right liver lobe (Couinaud segment 8). The patient was asymptomatic and had no remarkable medical history, including liver disease. He was not a habitual drinker or a smoker. A general examination that included the abdomen showed no particular findings. The laboratory data (complete blood count, chemistry, urinalysis, tumor markers, and coagulation) showed an elevated white blood cell count (11000/*μ*l) and CRP level (10.83 mg/dl). AFP, CEA, CA19-9, PSA, and PIVKA-II values were all within normal limits.

**Figure 1:**
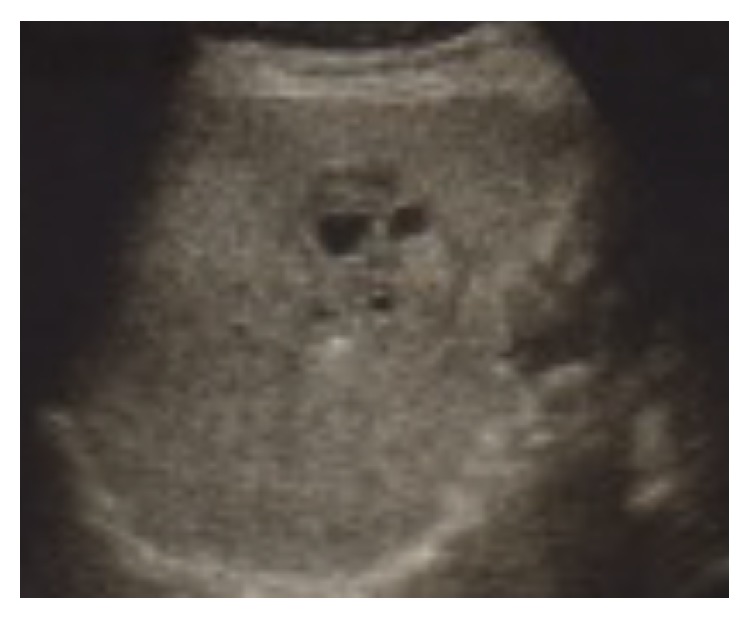
Ultrasonography (US) images. US showed an isoechoic nodule with small hypoechoic areas in segment 8 of the liver.

**Figure 2:**
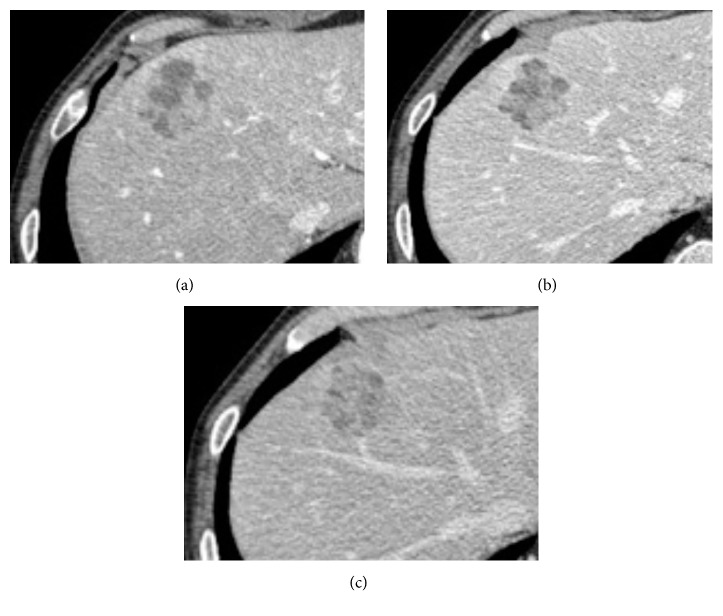
Images of contrast abdominal computed tomography (CT). CT showed that the tumor was slowly enhanced from the early phase to the parallel phase. (a) Early phase. (b) Delayed phase. (c) Parallel phase.

US showed an isoechoic nodule of 30 × 35 mm with hypoechoic areas in segment 8 of the liver (Figure 1). CT showed a tumor that was slowly enhanced from the early phase to the parallel phase (Figure 2).

Furthermore, drip-infusion-cholecystocholangiography-CT (DIC-CT) showed that there was no traffic between the tumor and the bile duct (data not shown).

**Figure 3:**
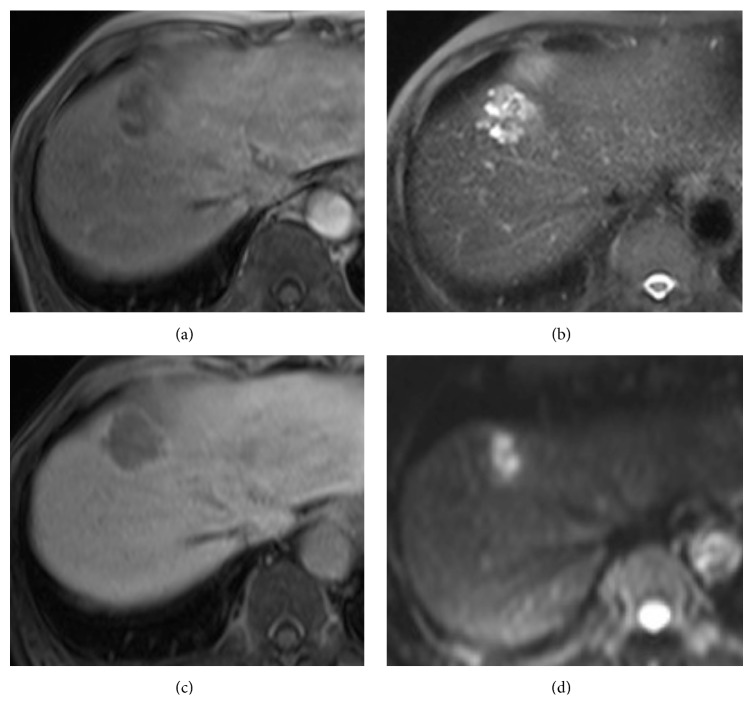
(a) T1-weighted magnetic resonance (MR) images showed the tumor with low intensity with lobulated borders. (b) The T2-weighted MR image shows the low signal portion indicating the cyst wall or bulkhead inside the high signal region. (c) In the hepatobiliary phase, the tumor showed low intensity. (d) On diffusion-weighted images, markedly high intensity was noted.

Magnetic resonance imaging (MRI) using Gd-EOB-DTPA contrast agent revealed a tumor with a high signal on T2-weighted imaging and a low signal on T1-weighted imaging accompanied by an enhancement effect on the cyst wall, and a bulkhead was found. Diffusion-weighted imaging revealed markedly high intensity (Figure 3). The patient had poor control of polymyalgia rheumatica and did not have a lesion other than on the liver, so the clinical decision was made to resect the mass for a diagnosis and treatment.

**Figure 4:**
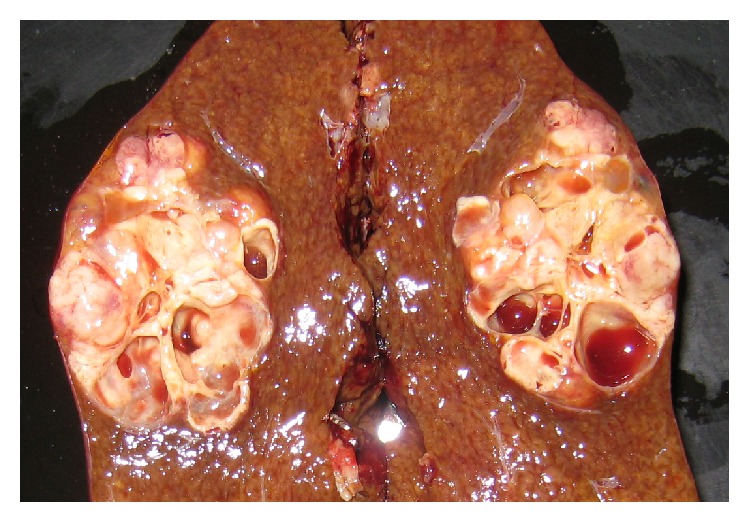
Resected specimen. The mass measured 35 × 50 mm and was located on the hepatic capsule.

Surgical resection of the anterior segment of the right hepatic lobe showed a 35 × 50 mm mass on the hepatic capsule. Tumor cleft was a mass with cystic structure in solid part (Swiss cheese-like); there was no necrosis inside (Figure 4).

## 3. Pathology

**Figure 5:**
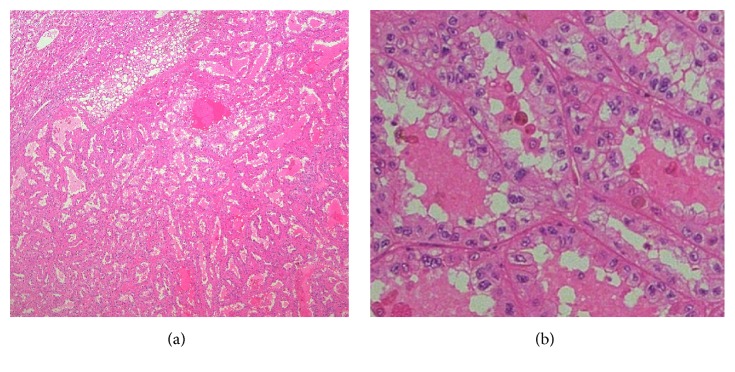
(a) The tumor comprised tubulocystic structured lesions (H&E). (b) Tumor cells have cell mutation with large nucleoli and eosinophilic cytoplasm, cubic and flat, forming small glands.

**Figure 6:**
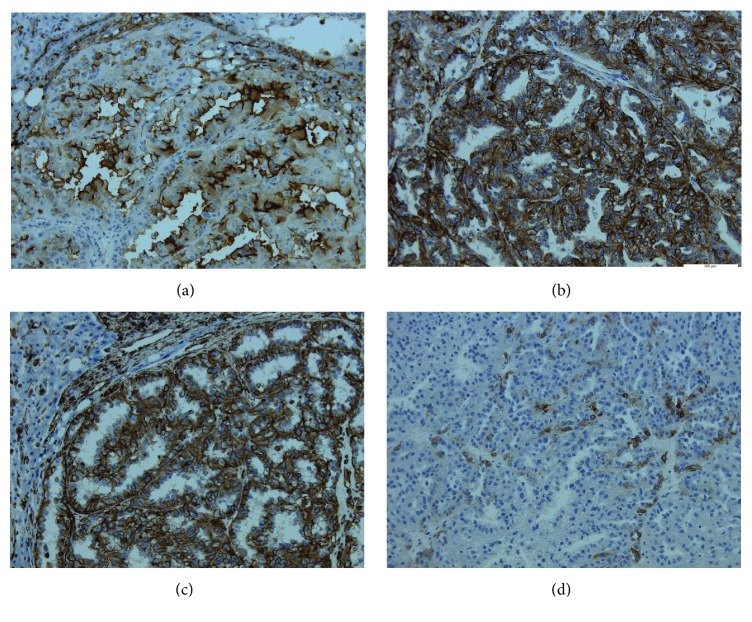
(a) CD10 positivity in the tumor cells. (b) CAM5.2 positivity in the tumor cells. (c) Vimentin positivity in the tumor cells. (d) CK7 being occasionally positive.

**Figure 7:**
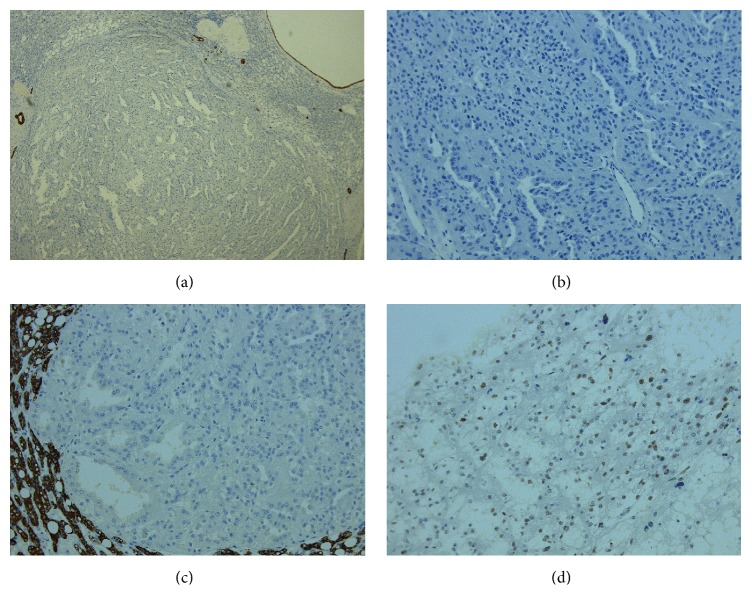
(a) AE1AE3 negativity in the tumor. (b) CD20 negativity in the tumor. (c) Hepatocyte negativity in the tumor. (d) p53 positivity in the tumor.

Histopathologically, when viewed at high-power magnification, tubular and tubulocystic structures were conspicuous, and these tubules/cysts were separated by thin fibrous stroma without any evidence of desmoplastic reaction. The tumor cells had large nucleoli and eosinophilic or amphotropic cytoplasm with mildly cellular variety, showing a cuboidal to flattened shape lining the tubules and microcysts (Figure 5). Immunohistochemically, these atypical epithelial cells were positive for CD10, cellular adhesion molecule (CAM) 5.2 (low-molecular cytokeratin), and vimentin, suggesting a biliary epithelial nature, and were occasionally positive for cytokeratin (CK) 7 (Figure 6). However, cytokeratin (clone: AE1AE3), *β*-catenin, progesterone receptor (PgR), thyroid transcription factor- (TTF-) 1, synaptophysin, thyroglobulin, surfactant apoprotein (SPA), epithelial-CMA (EpCAM), MUC5AC (a mucinous-associated protein), neural cell-CAM (NCAM), human melanin black (HMB) 45, and CD20 were negative (Figures 7(a) and 7(b)). On staining of hepatocytes (Figure 7(c)), alpha-fetoprotein (AFP), glypican-3, and arginase-1 were negative. In the tumor region, these carcinoma cells were specifically positive for p53 (Figure 7(d)).

Based on these findings, we made a conclusive diagnosis of tubulocystic carcinoma of the bile duct. The patient was well with no recurrence of the tumor in the liver at four-year follow-up after surgery.

## 4. Discussion

Most common biliary cystic tumors are of two types: biliary cystadenomas and adenocarcinoma. These are classified as either mucinous cystic neoplasm (MCN) or intraductal papillary neoplasm of bile duct (IPN-B) depending on the presence of ovarian stroma and bile duct communication (BDC), respectively [4].

Tubulocystic carcinoma (TCC) of the kidney is an exceedingly rare, recently recognized subtype of renal cell carcinoma and considered in differential diagnosis of cystic renal neoplasms. Diagnosis is based on distinct histological features combined with immune histochemical features. This case had histological and pathological features of tubulocystic carcinoma of the kidney. Only six cases of tubulocystic carcinoma that derived from the bile duct have been reported as subtype of the collecting duct carcinoma of the kidney in abstracts of the 102nd United States and Canadian Academy of Pathology (USCAP) annual meeting in 2013, and the details of the entity are unknown. Masson originally described tubulocystic carcinoma with Bellinian epithelioma as a tumor of the collecting ducts of Bellini in the kidney [5]. Therefore, an evolving concept of collecting duct carcinomas was proposed, and low-grade collecting duct carcinoma at the beginning of the spectrum corresponds to the current concept of tubulocystic carcinoma. Supportive findings include the expression of proximal convoluted tubule markers (CD10 and alpha-methylacyl-CoA racemase; AMACR), distal nephron proteins (parvalbumin, high-molecular-weight CK, and CD19) [1, 6], and the detection of intercalated cells and cells similar to those in the proximal tubule using electron microscopy [1, 7, 8]. In addition, Osunkoya et al. [8] reported that all tubulocystic carcinomas were strongly positive for the proximal convoluted tubule markers CD10, vimentin, and AMACR. Tubulocystic carcinoma exhibits a histopathological image that suggests a poor proliferative response from its cell heterotypes and structures but there are some cases with distant metastasis and local recurrence [1, 9, 10]. The pathological histology that predicts slow progression and dissociation of the clinical course leading to metastasis/recurrence is a feature of this disease.

Tubulocystic carcinoma of the bile duct is very unusual with regard to its histopathology, and no definite view has yet been obtained. We must carefully observe its clinical course as a nonclassifiable intrahepatic bile duct carcinoma. It may be recognized as a very rare tumor derived from the bile duct in the future.

## 5. Conclusion

We herein reported an extremely rare case of tubulocystic carcinoma that derived from the bile duct.
